# Post-Basic Meningitis

**Published:** 1909-03-20

**Authors:** F. J. Poynton

**Affiliations:** Assistant Physician to University College Hospital and to the Hospital for Sick Children, Great Ormond Street


### Hospital Clinics.

### POST-BASIC MENINGITIS.

By F. J. POYNTON, M.D., F.R.C.P., Assistant Physician to University College Hospital and to the Hospital for Sick Children, Great Ormond Street.

(A Lecture Delivered at University College Hospital.)

It is my intention in this lecture to deal with the clinical rather than the pathological side of post-basic meningitis; and in particular with some points in treatment which are of very considerable interest and importance.

First, nevertheless, it is essential that I should say a few words about the causation of this disease, which is also known as sporadic cerebro-spinal meningitis. In 1898 my colleague, Dr. Still, then Registrar at Great Ormond Street, described a non-Gram-staining diplococcus in the inflammatory exudations of post-basic meningitis; and it is now generally accepted that it is, as he believed, the causative agent of the disease. Later researches have shown that it is in many respects identical with the diplococcus described by Weichselbaum as the organism of epidemic cerebro-spinal fever. This in no way affects the value of Dr. Still's observations; and it is still doubtful whether post-basic meningitis is identical with the epidemic disease. Closely allied, no doubt, they are, but possibly not identical, that is not the result of precisely similar toxins. Even in the epidemic cases the organisms found show slight differences: in the recent outbreaks at Glasgow and Belfast the diplococcus isolated proved to be not quite the same as that of cases in London. Some differences have, for example, been noted in the sugars fermented by these meningococci, and in their staining reactions. Miss Alice Taylor is the observer who in this country has done the most work on these organisms, and I am much indebted to her for several hints as to the differences in behaviour of various meningococci. In a paper published in the *Lancet* on July 6, 1907, she contributed a study of the opsonic index and agglutination in cerebro-spinal meningitis; and she sent me a note quite recently to the effect that in post-basic meningitis the opsonic index and the agglutination of the meningococci alter altogether independently, whereas in the epidemic acute cases they rise and fall together. I am not competent to deal with these technicalities, and my object in alluding to them now is to direct attention to the important point that micro-organisms superficially alike may not necessarily produce the same disease in man, and that mysterious differences in them may have bearings upon treatment.

To sum up, then, it will serve our present purpose if we look upon post-basic meningitis as the result of an infection with a non-Gram-staining diplococcus. This is considered by some leading bacteriologists as identical with the diplococcus meningitidis of Weichselbaum; but we have, I think, good right to leave that an open question, and to be content with the view that the disease is very closely allied to the epidemic form without pinning ourselves down to their actual identity.

### Symptoms of Post-Basic Meningitis.

As to the disease itself, it nearly always attacks children under two years of age, and thus resembles pyopericardium. The onset is as a rule acute, and the three symptoms most frequently met with are vomiting, convulsions, and retraction of the head. Many cases are easy to diagnose when these three cardinal symptoms are associated. A physical sign of value is bulging of the anterior fontanelle, the result of increased intracranial pressure. To those of you who have not had particular opportunities in children's diseases I would emphasise the great importance of the condition of the anterior fontanelle in infants. In addition, there is often considerable pain, as evidenced by screaming and irritability; or, on the other hand, drowsiness, which may be ascribed to the toxæmia of the infection.

Retraction of the head is a most important sign, and is present in about half the cases on the first day of the illness, and in ten-elevenths before the end of the first week, according to the classical article by Sir Thomas Barlow and Dr. Lees in *Allbutt's System of Medicine*. Yet we must beware of thinking that retraction of the head in infancy is always a sign of meningitis, for it may occur in such different conditions as retro-pharyngeal abscess, acute pneumonia, otitis media, and erysipelas of the neck.

Another point is not to confuse head retraction with passive falling back of the head. In post-basic meningitis you will find both: retraction in the early stages, from spasm; and falling back, in the later paralytic stage. I have on more than one occasion seen cases of cerebral diplegia, in which there has been falling back of the head from paralysis of the cervical muscles, mistaken for meningitis, because this paralytic phenomenon has been interpreted as that spasmodic condition which is the cause of head retraction. Cerebral diplegia and post-basic meningitis are widely different diseases, and so the error is a very real one. The distinction is easy between retraction and falling back of the head. If you place the child on its abdomen the head is still retracted in spasm, but falls forward if there is paralysis.

### Ocular Symptoms.

Strabismus and oscillatory movements of the eyes—pseudo-nystagmus—are quite common: but alterations in the pupils are infrequent, and optic neuritis in particular is rare.

The next ocular symptom, blindness, is very common and reminds one forcibly of the similar phenomenon in epidemic cerebro-spinal fever. It is not due to disease of the optic nerve, but probably, as suggested by Barlow and Lees, to inhibition of the lower visual centres in the brain. This blindness may be a passing event, and if the child lives sight may be gradually recovered: weeks and months may elapse before this remarkable recovery is complete.

In only one case, and that about eight years ago, have I seen that inflammatory condition of the eyeball, which is known by ophthalmic surgeons as pseudo-glioma.

Other Symptoms.

Champing movements of the lower jaw and grinding of the teeth have often been recorded, and may be very persistent. Tonic spasm of the limbs may produce the most extraordinary deformities, especially when there are great head retraction and opisthotonus. This spasm may produce either flexion or extension.

Convulsions are frequent at the onset, but less so during the course of the disease. Paralysis is rare as compared with spasm. As the illness progresses "cyclical breathing," Cheyne-Stokes respiration, sighing, and yawning may be met with. The temperature is very variable and irregular, and hyperpyrexia is frequent as a terminal event, but quite exceptional otherwise. A chart of irregular fever in an infant with a vague illness suggests post-basic meningitis.

Rashes are met with as in the epidemic fever, but they are exceptional: they are usually erythematous. Arthritis is rare, but I have seen about half a dozen cases in all. One case, which I published in the *Edinburgh Medical Journal*, illustrates the rare event of arthritis as a prominent feature early in the illness: the patient was actually sent up for the arthritis alone. When this, as usual, occurs later it may not attract much attention. There may be considerable periarticular inflammation, and more rarely a copious effusion into the synovial cavity.

Constipation, as you may imagine, is often met with, as in most organic central diseases. Wasting is a very striking characteristic: it may reach to a most extreme degree, and you may be sure that the disease is getting the better of the patient if there is a steady fall in weight.

Lastly we come to hydrocephalus, which is generally recognised as one of the most serious events in the course of the disease. The cerebro-spinal fluid in the lateral and third ventricles drains through the iter into the fourth and escapes thence by the foramen of Majendie and the two foramina of Luschka into the sub-arachnoid space. These channels may become blocked by the inflammatory exudate, and thus hydrocephalus results.

It is essential in the proper management of a case of post-basic meningitis both to weigh the child regularly and to measure the maximum circumference of the head once a week, so that you may become apprised of the onset of hydrocephalus. This complication is a very grave one, carrying with it the danger of idiocy, epilepsy, and sudden death, which may occur most unexpectedly long after the original illness has subsided.

The course is variable, and may be very rapid. Death sometimes ensues in eight days, sometimes after many weeks. Sometimes recovery takes place slowly with or without hydrocephalus.

Diagnosis.

I do not intend to delay long over diagnosis, except to emphasise the great value of lumbar puncture. Before its introduction it was not always possible to differentiate tuberculous and post-basic meningitis, although optic neuritis, choroidal tubercle, and more rapid course are in favour of the former. So also is a more advanced age (over two), lesser degree of head retraction, and the presence of tuberculous foci elsewhere; but there are cases which are impossible of distinction, and defeat the most expert. One case of tuberculous meningitis in particular I can recall in which the inflammation was more than usually chronic, and also almost entirely post-basic in distribution. It ran a course of six weeks with great head retraction, and was unanimously considered to be simple post-basic meningitis. Another disease difficult to differentiate is polioencephalitis and anterior poliomyelitis.

For lumbar puncture asepsis is, of course, urgently needed. Use a small trocar and cannula; a fine inner tube for injecting serum is valuable. Just a hint or two from my observations upon unusual difficulties. The child is placed either on his face or on his side. The spine is flexed to separate the laminæ, and you must be sure that the spine is straight, not twisted. Locate the posterior superior spines of the ilia and note at what level the line joining them crosses the vertebral spines. Choose the intervertebral space next above this, a bare quarter-inch to one side of the mid-line. Insert the trocar and cannula through the skin and gently feel your way between the laminæ. Be careful not to hurry over this, or you may blunt your fine trocar against the bone. The direction is slightly upwards and a shade inwards towards the middle line. If you use a large trocar, you may fail, or have trouble with bleeding.

I have never myself seen any harm come from a lumbar puncture; but I should never lightly undertake it, nor dream of employing it without a very clear idea of what I wished to learn from it. In post-basic meningitis a more or less turbid fluid is obtained, which must be examined by an expert for meningococci, intracellular or otherwise: herein lies the great value of this procedure in diagnosis.

Treatment.

A great impetus has been given to the treatment of cerebro-spinal meningitis, whether sporadic or epidemic, by the remarkable results obtained with anti-meningococcic sera. The subject is one of particular interest, because at the present time conflicting results have been obtained in different outbreaks, and it is possible, as I have hinted, that, though in all cases we are dealing with some form of meningococcus, yet these may differ in character and reaction so far as serum is concerned as if they were different organisms.

A few words upon the history of anti-meningococcic sera. In 1906 Kölle and Wassermann satisfied themselves that by inoculating horses with living cultures of meningococci they were able to produce antibodies or protective substances in the serum. Jehrmann obtained virulent cultures and produced a serum from horses which, when injected direct into the spinal canal, gave favourable results.

Flexner has done very remarkable work in this direction. He reproduced the disease in monkeys, and by means of an homologous serum was able to bring about many recoveries. He then proceeded to treat the disease in human beings with an anti-serum obtained from horses, and gained a marked degree of success. In January 1908 Jobling and he reported 47 cases, with recovery in 34. In July the same writers tabulated 357 cases of the epidemic type, with numerous recoveries. Thus in 121 patients injected between the first and third days there were 103 recoveries. In 100 treated between the fourth and seventh days, 78 recoveries. In 107 treated after the seventh day, 68 recoveries. It may be added that the recovery was usually complete.

As perhaps you are aware, there was an epidemic of cerebro-spinal fever in Belfast in 1907-8, and Robb reports a most interesting result with Flexner's serum; of 30 patients treated 28 recovered, whereas the mortality before the serum was introduced was over 80 per cent. Epidemic cerebro-spinal meningitis is a curiously variable disease in the course it may run; but when one studies the scientific steps that have led to the production of Flexner's serum, the brilliant pathological investigations and the careful clinical records, it appears certain that one of the greatest advances in treatment has been achieved; and already an ample reward has been obtained.

This leads me next to Ruppel's serum, for the knowledge of which I am indebted to my friend Dr. Batten. I used it for post-basic meningitis in 1907 and 1908, because I could not get any of Flexner's, in three cases which I published last November in the "Transactions of the Royal Society of Medicine." In two of these I obtained remarkable results, and in the third I think I might have been more successful had I been more on the alert.

It would be wrong to claim on such scanty evidence any decisive proof of the action of this serum; and there is full proof that in the epidemic form of cerebro-spinal meningitis it has failed. Yet when we bear in mind that these two affections, though extremely closely allied, may not be absolutely identical, it is only fair to say that had I more opportunities I should most certainly give this serum a very complete trial in post-basic meningitis. In fact, for these sporadic cases, I should, with my present limited experience before me, try Ruppel's serum first, although quite prepared to use Flexner's if there is good evidence forthcoming of its value in these cases.

The steps to take are to confirm the diagnosis by lumbar puncture and bacteriological examination as soon as possible. The serum I should use is the dried form as sold by Lucius Brunner and Co., because with this I obtained the most striking result. In one case the serum was injected subcutaneously, and there was rapid recovery; in another the temperature fell several degrees, but death ensued; in the third subcutaneous injection had apparently no effect, but Dr. Jeffreys injected intraspinally on the third day afterwards, and complete recovery rapidly followed. The flakes of solid serum must be dissolved in sterile water.

At the discussion on these cases entire lack of success in six cases was reported by Dr. Torrens; but the serum used was not identical with that which we had. On the other hand, Miss Taylor wrote to me on November 15, 1908, saying that Dr. Fowler, in Edinburgh, on hearing from her of the successful result we had, treated a patient under his care by the same method, and obtained another success. This case is published in the *Journal of Neurology and Psychiatry* for January 1909, and is a very complete one. What one would like to see would be a careful trial of Ruppel's serum subcutaneously and intraspinally, with full reports. I should also like to try Flexner's serum, but have not yet had the chance.

There are one or two other points in treatment to which I may direct your attention. Cerebral vomiting is notoriously difficult, and often impossible, to stop; but I strongly advise an attempt, for sometimes it can be stopped more easily than one would think. Give food in small quantities, and peptonised; give also bismuth, which can at least do no harm. For pain and restlessness try small doses of chloral and bromides: of the former about one grain is the dose for a child a year old.

For constipation a small dose of calomel is indicated, and may be combined with a little Dover's powder if there is distress and irritability. A quiet room, tepid sponging if there is unusually high fever, and very careful management of the skin in the nursing of emaciated patients are all of importance.

The treatment of hydrocephalus is, in my experience, most disheartening. Every patient I have had operated upon has died, with one exception, and that was a child which succumbed a few months later to measles; and I could not persuade myself that the tube leading from the lateral ventricle to the sub-arachnoid space was of any service. On the other hand, the two most successful cases I have had were both allowed to run their course. The two children, now 14 and 12 years old respectively, are happy, well nourished, and able to get about quite well, though both are deficient in intelligence. Nevertheless there are a few successful cases on record where operation has been effective; for example, those reported by Sutherland and Cheyne, and by Lees and Ballance. If you are going to have an operation done, have it done early.

